# Comparative effectiveness of combined biologic agents versus standard therapies in the treatment of plaque psoriasis: a retrospective analysis

**DOI:** 10.3389/fmed.2024.1451069

**Published:** 2024-09-18

**Authors:** Bo Wu, Qian Chen, Rong Cao, Lei Zhu, Hongyan Zhu

**Affiliations:** ^1^Department of Dermatology, Children's Hospital of Soochow University, Suzhou, China; ^2^Department of Dermatology, The Affiliated Suzhou Hospital of Nanjing Medical University, Suzhou Municipal Hospital, Suzhou, China

**Keywords:** plaque psoriasis, biologic agents, therapeutic effectiveness, skin lesion resolution, adverse reactions

## Abstract

**Introduction:**

Plaque psoriasis is a persistent skin disorder that necessitates efficient management. This study investigates the therapeutic effectiveness and timeline for skin lesion resolution in plaque psoriasis patients treated with combined biologic agents compared to standard therapies.

**Methods:**

Conducted retrospectively between March 2020 and March 2023, the study included 162 patients with moderate to severe plaque psoriasis. Participants were divided into two groups: the Control Group, which received standard treatments, and the Combined Biologic Agent Group, which received additional biologic therapy with secukinumab. Participants in the Control Group received standard treatments, while those in the Combined Biologic Agent Group received standard treatments plus secukinumab.

**Results:**

The results showed that the Combined Biologic Agent Group experienced a significantly faster onset of therapeutic effects, with an average time of 3.04 ± 2.25 days compared to 6.12 ± 2.06 days in the Control Group. Additionally, skin lesion resolution occurred more rapidly in the biologic agent group (7.04 ± 2.13 days) than in the control group (14.56 ± 4.73 days). By week 24, the Psoriasis Area and Severity Index (PASI) scores demonstrated a more substantial reduction in the biologic agent group, decreasing from 26.98 ± 11.28 to 2.48 ± 3.01, whereas the control group showed a reduction from 25.82 ± 10.47 to 10.40 ± 7.63. The overall effectiveness rate was higher in the biologic agent group, with no cases of ineffectiveness, compared to a 20.99% ineffectiveness rate in the control group. Furthermore, there was no recurrence of the disease in the biologic agent group, while the control group experienced an 11.11% recurrence rate. Both groups had a similar incidence of adverse reactions, indicating that the addition of biologic agents does not significantly increase the risk of adverse events.

**Discussion:**

These findings suggest that combined biologic agent therapy offers a more effective and faster treatment option for plaque psoriasis without compromising safety. However, larger-scale clinical trials are necessary to validate these results and establish the long-term benefits and safety of this treatment approach in diverse patient populations.

## 1 Introduction

Plaque psoriasis is a common, long-lasting autoimmune skin condition characterized by excessive proliferation of skin cells, leading to the formation of thick, red, and scaly patches on the skin. This prevalent disorder has a profound negative influence on the quality of life for millions of individuals globally, causing major physical and psychological effects (1, 2). The physical discomfort, frequently accompanied by intense itching and pain, is accompanied by significant psychological misery, resulting in social shame and emotional difficulties. Managing plaque psoriasis remains a substantial challenge in clinical dermatology, despite extensive research and notable breakthroughs in dermatological therapies (3). The treatment options for plaque psoriasis have typically included a range of therapies, from applying medications directly to the skin to using medications that affect the entire body. For mild cases of psoriasis, topical therapies such as corticosteroids and vitamin D analogs are commonly used as the first line of treatment. Although these topical treatments are useful for treating localized and less severe symptoms, they often do not provide sufficient relief for moderate to severe conditions (4, 5).

In cases of greater severity, systemic medications such as methotrexate, cyclosporine, and acitretin have been the primary therapeutic approach. Methotrexate, an antimetabolite, decreases inflammation by impeding the rapid multiplication of skin cells. Cyclosporine, an immunosuppressive agent, functions by attenuating the hyperactive immune response that is characteristic with psoriasis. Acitretin, a retinoid that acts systemically, restores normal skin cell growth (6, 7). Although these systemic medicines are effective, they frequently come with substantial adverse effects and present hazards when used for extended periods. The combined toxicity, capacity to harm organs, and diverse side effects frequently require the termination or rotation of treatments, which makes long-term management solutions more complex. The constraints and negative characteristics of conventional systemic medicines have led to the investigation and rise of biologic drugs as novel treatment alternatives. These biologics, which are designed to specifically target certain parts of the immune system, have demonstrated encouraging outcomes in enhancing effectiveness and safety characteristics when compared to conventional treatments (8). These treatments function by specifically focusing on important cytokines or proteins on the surface of cells that are involved in the development of psoriasis, such as tumor necrosis factor-alpha (TNF-*α*), interleukin-17 (IL-17), and interleukin-23 (IL-23). This disrupts the inflammatory pathways that contribute to the progression of the disease (9).

The advent of biologics has been a pivotal milestone in the treatment of psoriasis, providing patients with improved management of their illness while minimizing adverse effects. However, this change in treatment strategy has led to new questions about the most effective ways to utilize these powerful agents, particularly in combination with traditional systemic therapies. This study specifically compares the effectiveness of a combined regimen of biologic and traditional systemic therapies versus traditional therapies alone in managing plaque psoriasis. Recent literature has inspired our study, and while the aforementioned studies provide crucial insights into the effectiveness and safety of biologics in treating psoriasis, our research also contributes significant new findings to the existing body of knowledge (10–12). Our research focuses on evaluating both the effectiveness and the safety profiles of these regimens in actual clinical settings, aiming to understand whether these combined approaches can enhance patient outcomes while also managing risks and costs effectively. The ultimate goal is to provide a detailed comparison that could inform future therapeutic strategies and optimize treatment outcomes for patients with plaque psoriasis.

## 2 Materials and methods

### 2.1 Study design

A retrospective assessment was conducted to evaluate the effectiveness of using a combination of biologic drugs in treating plaque psoriasis, as well as to determine the timing for resolving skin lesions. The retrospective analysis included the period from March 2020 to March 2023. The observation group for this trial consisted of 81 patients, comprising 18 pediatric patients and 63 adult patients, who were treated with a mix of standard therapies and biologic medicines. To conduct a comprehensive and accurate comparison, a control group consisting of an equal number of patients (*n* = 81) who received treatment over the same period was developed. However, the control group was differentiated by receiving solely conventional treatment methods, which ensured that there was a similar starting point between the two groups. Informed consent was obtained via telephone from all participants or their legal guardians prior to the inclusion of their data in the analysis. This process ensured that they were fully informed about the study’s objectives, methodology, and potential implications, aligning with ethical research practices. The study’s methodology and protocols underwent thorough review by our hospital’s ethics committee, and all methods were conducted in accordance with relevant guidelines and regulations, adhering to the ethical principles outlined in the Declaration of Helsinki. Data confidentiality was maintained, with all personal identifiers removed before analysis to protect participant privacy.

### 2.2 Inclusion and exclusion criteria

This study includes participants who have received a clinical diagnosis of moderate to severe plaque psoriasis from a certified dermatologist. Enrollment comprises individuals from various age groups, including both adults and children, at the moment they are diagnosed. It is essential that individuals who meet the requirements have had a combination therapy that includes conventional medicines such methotrexate, cyclosporine, or acitretin, as well as biologic drugs, for at least 12 weeks before starting this study. In addition, it is necessary for individuals to have a documented history of plaque psoriasis lasting a minimum of 6 months. Another important factor to consider is the stability of the disease. It is necessary for patients to have maintained a stable condition for a minimum of 1 month prior to their participation in the study.

To maintain a concentrated study cohort, patients with other kinds of psoriasis, such as guttate, inverted, pustular, or erythrodermic psoriasis, are not included in the study. Individuals who have severe cardiac, hepatic, renal, or hematological disorders are not allowed to participate if they simultaneously have other medical issues. Women who are pregnant, intending to conceive, or breastfeeding at the time of the research are ineligible. Moreover, any patient who has recently taken part in another clinical trial within the last 3 months will not be included in this study to prevent potential conflicts or influence from other experimental therapies or protocols.

### 2.3 Treatment protocols and follow-up

To ensure clarity and specificity in our study design, it is important to note that the Combined Biologic Agent Group received a treatment protocol that specifically included secukinumab as the only biologic agent, administered in combination with standard therapies. This protocol was chosen to evaluate the enhanced effectiveness of integrating secukinumab with established treatment methods for plaque psoriasis. The Control Group received standard treatment for plaque psoriasis, which included topical corticosteroids, vitamin D analogs, and phototherapy as indicated by disease severity and patient response. In contrast, the Combined Biologic Agent Group received an enhanced treatment protocol. In addition to the standard psoriasis therapies, these patients were administered subcutaneous injections of secukinumab. The dosing regimen involved an initial weekly injection of either 300 mg or 150 mg for the first 4 weeks, starting from week 0. This was followed by maintenance injections administered every 4 weeks, continuing until week 24. The dosing was tailored to the patient’s specific needs, with adjustments made based on their response and tolerance to the treatment. In cases where patients exhibited significant abnormalities or experienced severe adverse reactions, the biologic treatment was promptly discontinued to ensure patient safety. Following completion of the treatment course, all patients were monitored for 6 months post-discharge to assess long-term therapeutic effectiveness and the durability of skin lesion resolution.

### 2.4 Data collection and evaluation criteria in psoriasis treatment effectiveness

The primary objective of this study was to collect data and compare the clinical effectiveness, time of onset, timetable for resolution of skin lesions, and safety of the drugs utilized in two different groups. The assessment of clinical effectiveness was determined by analyzing the alterations in skin lesions and the Psoriasis Area and Severity Index (PASI) score. The effectiveness criteria were established in the following manner: Complete remission was determined by a decrease in the size of the lesion by at least 90%, the restoration of normal body temperature, and a PASI score of less than 3. Notable effectiveness was shown with a lesion decrease of at least 70%, normal body temperature, and a PASI score below 5. Partial effectiveness was defined as a decrease in the size of the lesion by at least 25%, occasional occurrence of fever, and a PASI score below 7. Treatment was deemed ineffective if there were no substantial alterations in lesions, persistent fever, and no modification in the PASI score. The definition of lesion healing was assessed based on three primary criteria: (1) Complete healing was quantified by a decrease in the size of the lesion by at least 90%, as measured by physical examination and/or photographic documentation at each follow-up visit. (2) Healing was also assessed based on the restoration of normal skin texture and integrity without scales or plaques, which was evaluated during physical examinations. (3) The PASI score, a widely recognized measure in dermatological studies, was utilized to objectively assess and quantify healing. A PASI score of less than 3 was considered indicative of complete lesion healing. The overall effectiveness rate was determined by dividing the sum of cases with complete remission, considerable effectiveness, and partial effectiveness by the total number of cases, and then multiplying the result by 100%. In addition, the study recorded the duration it took for the medicine to take effect and for the lesions to heal in all groups of patients. The frequency of disease recurrence and the occurrence of adverse events were also documented for each group.

### 2.5 Statistical analysis

Statistical analyses were rigorously conducted using SPSS software (Version 27.0). Data were initially categorized as quantitative or categorical, and normality tests were applied to determine their distribution patterns. For quantitative data that adhered to a normal distribution, inter-group comparisons were performed using independent sample t-tests, with results expressed as mean ± standard deviation. Categorical data were represented as frequencies and percentages, and their associations were evaluated using Chi-square (*χ*^2^) tests. All statistical hypotheses were tested two-tailed, with a *p*-value of less than 0.05 considered indicative of statistical significance.

## 3 Results

### 3.1 Baseline characteristics and clinical presentation

The study included a total of 162 patients, evenly divided into the Control Group (*n* = 81) and the Combined Biologic Agent Group (*n* = 81). The baseline characteristics of the two groups were well-matched, with no statistically significant differences observed in age, gender distribution, body mass index (BMI), age at diagnosis of psoriasis, or duration of the disease. The prevalence of psoriatic arthritis and previous treatments for psoriasis were also similar between the two groups. Both groups exhibited comparable PASI scores, indicating similar disease severity at baseline. The Body Surface Area (BSA) affected by psoriasis was also similar between the groups. Additionally, a similar proportion of patients in both groups reported worsening of psoriasis symptoms after stress. In terms of clinical presentation, the degree of pruritus and the types and colors of lesions were equally distributed across the two groups, with no significant differences. These findings suggest that the Control and Combined Biologic Agent groups were comparable at baseline, providing a reliable foundation for subsequent analysis of treatment outcomes (Table 1).

**Table 1:** Baseline characteristics of the control group and combined biologic agent group.

Characteristics	Control group (*n* = 81)	Combined biologic agent group (*n* = 81)	*χ*^2^/*t* value	*P* value
Age (years)	47.15 ± 11.50	45.29 ± 10.98	1.053	0.294
**Gender**				
Male/Female	60/21	62/19	0.033	0.855
BMI (kg/m^2^)	25.18 ± 3.06	24.95 ± 3.30	0.460	0.646
Age at diagnosis of psoriasis (years)	30.89 ± 13.59	31.89 ± 11.60	0.504	0.615
Psoriasis duration (years)	14.55 ± 9.89	15.43 ± 10.14	0.559	0.577
Psoriatic arthritis, *n* (%)	9 (11.11)	8 (9.88)	0.066	0.768
Previous psoriasis treatment, *n* (%)	48 (59.26)	45 (55.56)	0.227	0.634
PASI score	25.82 ± 10.47	26.98 ± 11.28	0.678	0.496
BSA affected (%)	33.52 ± 18.65	35.11 ± 21.94	0.497	0.620
Psoriasis worsened after stress, *n* (%)	49 (60.49)	46 (56.79)	0.229	0.632
Degree of pruritus, *n* (%)			0.245	0.876
Mild	42 (51.85)	41 (50.62)	/	/
Severe	39 (48.15)	40 (49.38)	/	/
Types of lesion, *n* (%)			0.234	0.629
Plaque	48 (59.26)	51 (62.96)	/	/
Mix (plaque + papule)	33 (40.74)	30 (37.04)	/	/
Color of lesion, *n* (%)			0.222	0.637
Red	39 (48.15)	42 (51.85)	/	/
Pink	42 (51.85)	39 (48.15)	/	/

BMI, body mass index; PASI, psoriasis area and severity index; BSA, body surface area.

### 3.2 Therapeutic onset and lesion resolution in plaque psoriasis treatment

In this study, notable differences were observed in both the therapeutic onset and the timeline for skin lesion resolution between the control group and the group treated with combined biologic agents. The control group had a longer onset of therapeutic effect, with an average time of 6.12 ± 2.06 days. In contrast, the combined biologic agent group demonstrated a significantly shorter onset time, averaging 3.04 ± 2.25 days. Similarly, the resolution of skin lesions was markedly faster in the combined biologic agent group, with an average resolution period of 7.04 ± 2.13 days, compared to 14.56 ± 4.73 days in the control group. At week 24, the PASI score for the control group was significantly reduced to 10.40 ± 7.63 from a baseline of 25.82 ± 10.47. In the combined biologic agent group, the PASI score showed an even more pronounced decrease, from 26.98 ± 11.28 at baseline to 2.48 ± 3.01 at week 24.

### 3.3 Clinical effectiveness of treatment modalities in plaque psoriasis

The results of the study reveal significant differences in clinical effectiveness between the control group and the group treated with combined biologic agents in the management of plaque psoriasis. The overall effectiveness, which encompasses complete remission, significant effectiveness, and partial effectiveness, was markedly higher in the combined biologic agent group compared to the control group. In the combined biologic agent group, the rate of complete remission and significant effectiveness combined accounted for over 86% of the patients (70 out of 81), illustrating a substantial impact of this treatment modality on improving patient outcomes. Notably, this group also reported no cases of ineffectiveness, underscoring the potential of combined biologic agents in effectively managing symptoms of plaque psoriasis. Conversely, in the control group, the total effectiveness rate was 79.01%, with a notable 20.99% of patients showing ineffectiveness in treatment. The distribution of effectiveness across categories in this group indicates a more moderate response to the treatment, highlighting the limitations of traditional therapy methods in some patient populations. These findings suggest a significant superiority of combined biologic agents over traditional treatment methods in terms of achieving complete remission and overall treatment effectiveness in plaque psoriasis. The distinct contrast in treatment outcomes between the two groups underscores the potential benefits of integrating biologic agents into treatment protocols for more effective management of this chronic skin condition (Table 2).

**Table 2:** Comparison of clinical effectiveness between control and combined biologic agent groups.

Group	Complete remission	Significant effectiveness	Partial effectiveness	Ineffective	Total effectiveness
Control group (*n* = 81)	12 (14.81%)	23 (28.40%)	29 (35.80%)	17 (20.99%)	64 (79.01%)
Combined biologic agent group (*n* = 81)	29 (35.80%)	41 (50.62%)	11 (13.58%)	0 (0.00%)	81 (100.00%)

### 3.4 Recurrence and adverse reaction analysis in plaque psoriasis treatment

A notable observation from the study was the absence of recurrence in the combined biologic agent group, with a 0% recurrence rate, compared to an 11.11% recurrence rate in the control group. This significant difference underscores the effectiveness of combined biologic agents in sustaining remission of symptoms. Regarding adverse reactions, both groups exhibited a similar total incidence rate of 14.81%. However, the distribution of specific adverse reactions differed. The control group did not report any cases of hypertension, while the combined biologic agent group had a 3.70% incidence rate in this category. Both groups had comparable rates of dyslipidemia, hepatic dysfunction, and renal dysfunction, each recorded at 3.70%. The similarity in the total incidence of adverse reactions between the two groups, despite differences in specific categories, suggests that while combined biologic agents significantly reduce recurrence, they do so without substantially increasing the overall risk of adverse reactions compared to traditional treatment methods (Table 3).

**Table 3:** Comparison of recurrence rates and adverse reactions between control and combined biologic agent groups.

Group	Recurrence rate	Hypertension	Dyslipidemia	Hepatic dysfunction	Renal dysfunction	Total incidence
Control group (*n* = 81)	9 (11.11%)	0 (0.00%)	3 (3.70%)	6 (7.41%)	3 (3.70%)	12 (14.81%)
Combined biologic agent group (*n* = 81)	0 (0.00%)	3 (3.70%)	3 (3.70%)	3 (3.70%)	3 (3.70%)	12 (14.81%)

## 4 Discussion

This study provides a unique insight into the comparative effectiveness of combined biologic agents versus standard therapies in the management of plaque psoriasis. By integrating a retrospective analysis over a three-year period, the research meticulously evaluates the accelerated therapeutic onset and enhanced resolution timeline of skin lesions using secukinumab in conjunction with standard therapies. This approach is distinct because it isolates the effects of a single biologic agent within a combined treatment regimen, providing a clear contrast to conventional treatment methods alone. The clinical value of this study lies in its rigorous assessment of secukinumab as a pivotal component in the treatment landscape for moderate to severe plaque psoriasis. The findings demonstrate a significant reduction in the time to therapeutic effect and a quicker resolution of lesions, which are crucial metrics for improving patient outcomes in chronic conditions like psoriasis. Moreover, the study adheres to stringent ethical standards and robust methodological protocols, ensuring the reliability and applicability of the results to a diverse patient population. The results of this study advocate for a strategic reevaluation of treatment protocols in dermatological practice, particularly for patients with persistent or severe plaque psoriasis. By providing evidence of the superior effectiveness of a combined regimen involving secukinumab, the study supports the integration of biologic agents with traditional therapies to achieve optimal control of the disease. This could lead to tailored treatment approaches that consider the specific needs and response patterns of individual patients, potentially enhancing the quality of life and long-term disease management for those affected by plaque psoriasis.

Plaque psoriasis, a persistent inflammatory skin condition, poses a considerable difficulty in terms of treatment, typically requiring long-term management techniques. The complex nature of the illness, which involves immunological dysregulation and alteration of the epidermal barrier, requires a strategy that goes beyond typical therapeutic methods (13). Biologic drugs have greatly transformed the treatment of psoriasis by providing focused therapies that target specific cytokines and inflammatory pathways involved in the development of the disease. Nevertheless, the diversity of patient reactions and the limited effectiveness reported in certain individuals emphasize the necessity for more complete treatment approaches. The role of combination biologic medicines is especially relevant in this context (14, 15). By combining numerous biologics that target distinct parts of the immune response, there is the potential for a comprehensive and more efficient approach to managing the disease. Jung et al.’s (10) study corroborates the rapid effectiveness of secukinumab in managing moderate-to-severe psoriasis, surpassing ustekinumab with a similar safety profile. Unlike their findings of allergic reactions and tuberculosis, our research indicates a potentially safer profile for secukinumab, enhancing its suitability as a preferred biologic therapy. Our analysis extends beyond individual biologic effectiveness by comparing these treatments directly with standard therapies, revealing superior effectiveness and a comparable safety profile of secukinumab, which supports its broader integration into clinical protocols. Valenti et al.’s (11) long-term data on ixekizumab, showing substantial PASI score improvements, align with our observations but our study provides a broader comparative context. Similarly, Fiorillo et al.’s (12) work on severe psoriasis underscores the sustained effectiveness of biologics, consistent with our findings across varying severities. Collectively, our research advocates for the expanded use of biologics like secukinumab in clinical practice, promoting a personalized, effective management strategy for psoriasis that spans all severity levels, potentially setting new standards in therapeutic protocols.

Including body temperature as a criterion for evaluating treatment effectiveness provides crucial insights into the systemic inflammatory status associated with moderate to severe plaque psoriasis. This measure helps assess the comprehensive impact of treatments, notably biologic agents, on both skin lesions and systemic inflammation, highlighting their dual effectiveness. The normalization of body temperature post-treatment indicates the anti-inflammatory success of therapies like secukinumab, supporting their role in holistic psoriasis management that addresses both dermatological and systemic health aspects. The results demonstrate a significant differential impact of combined biologic, and standard therapies compared to standard therapies alone for treating moderate to severe plaque psoriasis. The combination treatment, specifically integrating secukinumab, markedly improved therapeutic onset and lesion resolution, reducing PASI scores from 26.98 to 2.48 within 24 weeks, signaling potential long-term remission. Such effectiveness not only underscores the potent anti-inflammatory and immunomodulatory effects of secukinumab but also supports its integration into treatment regimens without increasing adverse reactions like hypertension or dyslipidemia (16). This aligns with literature advocating biologics for sustained disease control and modifies traditional treatment paradigms to enhance patient outcomes.

The significantly quicker therapeutic onset and eradication of lesions in the group treated with a combination of biologic agents indicates a more effective regulation of the pathophysiological pathways that cause plaque psoriasis. Biologic drugs, which are specifically engineered to target particular cytokines and pathways that play a role in the inflammatory process, are expected to offer a more precise and swift inhibition of the inflammatory cascade. This can elucidate the expedited enhancement and reduced duration for the resolution of skin lesions. Prompt and effective response to treatment is essential for managing symptoms, minimizing the physical and psychological strain on patients, and enhancing compliance with therapy (17). The enhanced clinical success of the combination biologic medicines, seen by increased rates of complete remission and notable effectiveness, can be attributable to the synergistic impact of targeting numerous pathways in the immune response. These medicines May offer a more comprehensive management of the disease process by simultaneously influencing several cytokines such as TNF-*α*, IL-17, and IL-23. This comprehensive strategy tackles the intricate causes of plaque psoriasis, which frequently involve different types of immune cells and communication molecules. The lack of recurrence in the group treated with a combination of biologic agents is especially remarkable (18, 19). This finding May be attributed to the continuous regulation of the immune system, which prevents the usual pattern of improvement and worsening observed in plaque psoriasis. The persistent impact of this action is crucial for the management of diseases over an extended period and enhancing the well-being of patients, as maintaining consistent control over the condition is a significant obstacle in the treatment of psoriasis.

The comparable occurrence of negative side effects in both treatment groups, despite variations in specific categories of adverse reactions, indicates that the use of combination biologic medicines does not dramatically elevate the risk profile when compared to traditional therapy. Nevertheless, the noted rise in hypertension within the biologic agent group necessitates additional examination. This May be attributed to the mechanism of action of these drugs or an unexpected interaction within the intricate immunological pathways. Continual monitoring and surveillance after a product are on the market are necessary to completely understand these safety factors. The ramifications of these findings are substantial for the therapy of plaque psoriasis. Firstly, they endorse the incorporation of combination biologic medicines into treatment protocols, especially for patients who have not achieved satisfactory outcomes with conventional therapy. Furthermore, the study emphasizes the necessity of tailored treatment strategies that consider the unique reactions and risk profiles of each individual patient. Ultimately, the findings emphasize the significance of a comprehensive therapeutic approach that not only prioritizes symptom regulation but also considers the long-term management of the condition and the overall quality of life.

This study has several limitations. The retrospective design of the study May introduce selection bias due to its reliance on past data, which May not accurately capture all variables. Furthermore, the sample size, while sufficient, restricts the applicability of the findings to a wider population. Furthermore, the lack of a comprehensive long-term follow-up hinders our comprehension of the lasting effectiveness and safety of combination biologic therapies. In addition, the study failed to consider potential confounding variables such as lifestyle, genetic predispositions, and environmental influences that could impact the results. The study did not include particular information about the characteristics and severity of plaque psoriasis in the participants, which May affect how applicable the results are to all people with plaque psoriasis.

In practice, incorporating biologics with traditional therapies offers a strategic advantage, particularly for patients unresponsive to conventional treatments alone. This study endorses a personalized approach, tailoring treatments to individual tolerability and disease response, which May revolutionize management strategies in dermatology. Our findings advocate for broader adoption of combined therapy protocols, potentially setting a new standard of care for specific patient demographics. Future investigations should focus on the long-term benefits of these regimens, especially their impact on quality of life and maintenance of remission, further defining the strategic role of biologics in psoriasis management.

## 5 Conclusion

This study demonstrates that combined biologic agent therapy provides superior effectiveness in the treatment of plaque psoriasis, evidenced by a more rapid onset of action and a significantly shorter timeline for skin lesion resolution, along with a more pronounced reduction in PASI scores. Notably, this enhanced therapeutic benefit does not correspond with a significant increase in adverse reactions, suggesting a favorable safety profile. However, to confirm these results and fully establish the broader benefits and safety of this therapeutic approach, larger-scale clinical trials are needed across diverse patient populations.

## Data Availability

The raw data supporting the conclusions of this article will be made available by the authors, without undue reservation.
